# The Development of Morphological Knowledge and Spelling in French

**DOI:** 10.3389/fpsyg.2020.00146

**Published:** 2020-02-13

**Authors:** Ruth Mussar, Monique Sénéchal, Véronique Rey

**Affiliations:** ^1^Department of Psychology, Carleton University, Ottawa, ON, Canada; ^2^INSPé (institut de formations des enseignants)-AMU, Aix-Marseille Université, Marseille, France

**Keywords:** spelling, silent letters, morphology, development, grades 1–3

## Abstract

The Frenchorthographic system is particularly difficult to learn because nearly 30% of words in the lexicon end with a silent letter. One metalinguistic skill that has been identified to facilitate spelling acquisition in French is morphological knowledge. This cross-sectional study investigated the construct of morphological knowledge, its development and its role in building accurate orthographic representations in a sample of francophone elementary students. We proposed that morphological knowledge, a superordinate process, encompasses children’s implicit use of morphemes in everyday language and their conscious, targeted manipulation of morphemes. In the present study, we assessed children’s recognition of morphogrammes, the silent-letter endings (SLEs) of root words that become pronounced in suffixed forms (e.g., the silent t in chant/ʃã/ [song] → chanteur /ʃãtœʀ/ [singer]). When spelling root words, children may mark morphogrammes by recalling morphologically related words in which the morphogramme is not silent – thus, morphological knowledge was hypothesized to positively predict morphogramme spelling. One hundred and twenty-three children in grades 1–3 were assessed on four measures of morphological knowledge, two measures of spelling recognition and a dictation of pseudowords to explore their inclusion of silent-letter endings in novel words. As expected, morphological tasks that required explicit morphological manipulations were harder than implicit ones. Moreover, first graders struggled to complete explicit morphological tasks, while third graders were near ceiling performance on implicit tasks. Nevertheless, the four tasks converged on a single morphological knowledge construct as confirmed by a factor analysis. Importantly, morphological knowledge explained unique variance in children’s accurate representation of silent-letter endings after controlling for grade, reading for pleasure and general orthographic recognition of words. Finally, children rarely used silent-letter endings when spelling pseudowords; however, when they did, they displayed sensitivity to the appropriate phonological context for the letter used. The findings are in accord with theoretical models suggesting that the representations of letters without phonological value are difficult to construct and may remain fuzzy.

## Introduction

Written languages that use an alphabet mark the phonology of spoken languages. The degree of correspondence between phonology and orthography, however, varies across these languages. Some languages, like Italian, are described as transparent because of the high consistency between phonemes (i.e., spoken sound) and the corresponding graphemes (i.e., letters or group of letters). Other languages, like English and French, are described as opaque because of the low consistency in phoneme-grapheme correspondences. Consider that only 21% of French words can be spelled by sound alone (Ziegler et al., 1996). This lack of phoneme-grapheme consistency in the French orthographic lexicon is caused by at least two factors. First, inconsistency arises when a phoneme can be spelled in numerous ways (Véronis, 1986). For example, the French nasal vowel /ɛ̃/ can be spelled in eight different ways: in (*lapin* [rabbit]), im (*timbre* [stamp]), ain (*main* [hand]), aim (*faim* [hunger]), en (*examen* [exam]), ein (*peinture* [paint]), yn (*lynx*) or ym (*thym* [thyme]). The second factor is the presence of letters without phonological value, silent letters that frequently occur at the end of words (Jaffré and Fayol, 2006). For example, the final letter of each of these words is silent: *boue /bu/* [mud], *chant* /ʃɑ̃/ [song], *chaud* /ʃo/ [hot], *gros* /gʁo/ [large] and *prix* /pʁi/ [price]. The present study investigated how young elementary-school children build accurate orthographic representations of French words that end with silent letters.

It is estimated that 56% of words present in children’s school books in France contain silent-letter endings (SLEs) – endings that most often encode grammatical or semantic information (Lété et al., 2004; Gingras and Sénéchal, 2017). After removing inflected words (i.e., the silent plural and conjugated forms), Gingras and Sénéchal still found that 29% of words ended with a silent letter. In these remaining words, SLEs often become pronounced in morphological derivatives (e.g., chant /ʃɑ̃/ [song], chanter /ʃɑ̃te/ [to sing] and chanteur /ʃɑ̃tœʁ/ [singer]). In other cases, SLEs help distinguish homophones (e.g., sang [blood] and sans [without] are both pronounced /sã/), or they indicate an idiosyncratic spelling (léotard/leotaʁ/ [leotard]).

By virtue of their silent nature, SLEs cannot be easily conveyed through speech. This, in turn, makes it difficult for children to learn these endings. Even after 5 years of schooling, children still have more difficulty spelling words with silent letters as compared to those that do not (Gingras and Sénéchal, 2019). Conducting stringent analyses, Gingras and Sénéchal confirmed the detrimental effect of SLEs to spelling accuracy after controlling for well-established predictors such as word frequency, word length, phoneme-grapheme consistency and rime consistency. Given this particular difficulty, it becomes of interest to understand how children come to mark silent letters over and above rote memorization.

The nature of the French language is such that silent letters often convey morphological information. Lexical morphogrammes are silent letters at the end of root words that are pronounced in suffixed words of the same word family (Catach, 1995). In the previous example, the silent t in *chant* is pronounced in the derived *chanteur*. Given the frequency of occurrence of morphogrammes in French, researchers have investigated whether children’s morphological knowledge would facilitate their spelling accuracy of SLEs. That is, if children think about words in terms of such morphological relations, then they may recall when a word contains an SLE and what that silent letter is. Previous findings have shown that children’s understanding of morphemes contributes to their early spelling (Sénéchal et al., 2006; Desrochers et al., 2018). To better understand spelling acquisition, it therefore becomes important to understand the development of morphological knowledge.

Herein, morphological knowledge refers to the superordinate process encompassing children’s morphological awareness and their morphological processing. Morphological awareness refers to the ability to consciously recognize and manipulate morphemes (Kuo and Anderson, 2006; Lam and Chen, 2017). This includes being able to recognize and segment the subcomponents of words and use these components to create novel words to fit a context. By contrast, morphological processing refers to the implicit ability to use morphemes for everyday language production (Kruk and Bergman, 2013; Nagy et al., 2013). For example, 4- and 5-year-old children, at an implicit level, recognize the relation between verbs and the agentive suffixes -er and -ist (Clark and Cohen, 1984). The child may even create new words using these suffixes. However, the child does not yet have explicit morphological awareness because they may not be able to explain the function of the suffix -er or -ist.

Although children do develop some morphological awareness prior to formal literacy instruction (Clark and Hecht, 1982; Casalis and Louis-Alexandre, 2000; Kim, 2011), it is not until they enter school that a substantial shift in children’s morphological knowledge takes place. With sufficient exposure to print, children begin to recognize how certain words contain common and specific visual elements, in addition to common elements of sound and meaning (Sénéchal and Kearnan, 2007; Pacton and Deacon, 2008; Abbott et al., 2016). Children can then merge these three components, allowing for more complex morphemic analysis and decomposition of multimorphemic words; in turn, this may enable children to read more complex material, further increasing their exposure to novel words (Berninger et al., 2009; Goodwin and Ahn, 2010). In other words, even as they learn to associate oral vocabulary with written symbol sequences, they begin to recognize how certain smaller sequences provide unique meaning (Carlisle, 2010; Manolitsis et al., 2019). By first grade, children can explicitly derive transparent, high-frequency derivational suffixes (Anglin et al., 1993; Carlisle and Nomanbhoy, 1993). By second grade, children possess sufficient relational knowledge to explicitly identify how some words share phonological, orthographic and semantic components beyond simple grammatical features; they are also capable of synthesizing or segmenting common morphemes (Casalis and Louis-Alexandre, 2000). By third grade, children can, albeit with difficulty, use their understanding of affixes to compose morphologically appropriate non-words to fit a specific context (Duncan et al., 2009).

These rapid changes in young children’s capabilities reveal the development of increasingly sophisticated and abstract cognitive processes. Consequently, when studying participants from different cohorts, multiple morphological measures may be needed to assess both the range of behaviours children can perform and the depth to which said behaviours can be performed. For example, relational judgement tasks, wherein participants are asked to determine if two phonologically similar words are semantically related, are generally considered easy, as they can ultimately be solved using implicit morphological processing (i.e., a “gut-feeling” that the two targets are related). This type task is often considered appropriate for younger children as it requires little in the way of working memory or cognitive effort and no explicit morpheme manipulation on the part of the child (Colé et al., 2004; Duncan et al., 2009). However, older children may reach ceiling performance, limiting variability in their scores; for example, Duncan et al. (2009) observed grade 3 students complete this task with upwards of 80% accuracy. By contrast, a decomposition task requires children to successfully identify the root and affix of a word and then isolate said root or affix. This task relies on children’s ability to explicitly manipulate morphemes, although implicit knowledge may also play a role, as children may be “primed” by hearing the root within the derived form. Analogy tasks are again more difficult, requiring children to recognize a morphological relation in one word pair, and apply this relation to complete a second word pair; for example, *haut* [high]: *hauteur* [height]∷ *gros* [large]: ______ *grosseur* [size]. In this task, the first item in the pair is a root with a silent morphogramme that becomes pronounced in the derived item. These tasks require both explicit morpheme production and a sophisticated level of reasoning – Sénéchal (2000) and Casalis et al. (2011) both found that grade 4 students only perform this task at approximately 65% accuracy. Though some argue that analogy tasks are inappropriate for younger children (see Kirby et al., 2012,) others have found some success with their implementation (e.g., Desrochers et al., 2018).

Current models of orthographic learning suggest that children begin to learn to spell by first associating individual letters with sounds, and through print exposure during reading, eventually refining their representations to whole word patterns (Ouellette, 2010; Sanchez et al., 2012; Conrad et al., 2013). The practice of spelling provides its own positive feedback loop – as children attend to each letter of the target word and the sound associated with it, their mental representation of the word strengthens (Ouellette, 2010). However, when a grapheme is phonologically underspecified, children’s representations of it may be “fuzzy” (Sénéchal et al., 2016). Consequently, when faced with a phonologically underspecified grapheme, such as a silent letter, children may not accurately represent what that letter is, if they represent it at all (Sénéchal et al., 2016; Gingras and Sénéchal, 2019). In one study of children in grades 1–3, approximately 63% of children’s SLE spelling errors in root words were the omission of the SLE, while the remainder were substitutions (Sénéchal et al., 2016). It is therefore of interest to understand how children overcome this difficulty. French maintains the principle of root consistency, meaning that the graphemes of root words are maintained in derivative forms. Though unpronounced (and underspecified) in the root, SLEs become salient in derived forms. Recalling derivatives forms of a root thus allows children to encode otherwise silent morphogrammes – for example, children can mark the silent “t” in *chant* [singing] by recalling its derivative *chanteur* [singer] (Sénéchal et al., 2006, 2016; Fejzo, 2016).

As an alternative to morphological awareness, it could be argued that words with large families benefit from orthographic redundancy, and that children recall SLEs based on orthographic relatedness. Support for a morphological explanation, rather than an orthographic one, was found in two studies. First, grade 4 children who reported using a morphological strategy (i.e., thinking of a derived word) spelled morphological root words with SLEs as accurately as did children who reported using retrieval, and morphological strategy users were more accurate than phonological strategy users (Sénéchal et al., 2006). Second, a study by Pacton et al. (2018) also provided evidence for the notion that it is morphology, not orthographic relatedness, that is the explanatory factor. In their research, grade 3 and 5 children were exposed to pseudowords with SLEs when reading texts that explained their meaning. In the morphological condition, the text also included two plausible derivatives that revealed the morphogrammes, whereas in the orthographic condition, the text included two orthographically related words that revealed the SLE but for which the suffix was implausible. In both conditions, the text included an opaque pseudoword that ended with a different SLE. Across conditions, children were matched on reading and spelling skills. Children in the morphological condition spelled more pseudowords accurately than opaque words, whereas children in the orthographic condition did not (also see, Pacton et al., 2013).

Correlational evidence also supports a significant role for morphological knowledge. Sénéchal (2000) showed, in 122 grade 2 and 4 children, that morphological knowledge accounted for unique variance in spelling roots with SLE morphogrammes after controlling for grade, general spelling, print exposure, oral vocabulary and phoneme awareness. This effect was specific to roots with SLE morphogrammes because morphological knowledge was not a significant contributor to spelling root words for which the SLEs were not morphogrammes (e.g., foulard /fulaʁ/ [scarf]). This specificity of the contribution of morphological knowledge to spelling SLE morphogrammes, as opposed to words with SLEs that are not morphogrammes, was replicated in a small sample of grade 4 children (Sénéchal et al., 2006). Fejzo (2016) provided further evidence of the specific role played by morphological knowledge in spelling French words. In this study, 75 children in grades 3 and 4 were asked to spell 31 complex words containing prefixes, bases with inconsistent graphemes, morphogrammes or suffixes. Children’s spelling accuracy was assessed at the whole word level and at the level of individual morphemes. Morphological knowledge was assessed using real and pseudoword derivation tasks. Hierarchical regressions revealed that morphological knowledge predicted 4% of variance in spelling morphogrammes and 9% of variance spelling suffixes after controlling for grade, word identification, non-verbal intelligence and phonological awareness. By contrast, morphological knowledge did not predict whole word spelling once morpheme spelling was added to the model – thus, showing the specificity of the effect. In summary, the available evidence converges to a specific effect of morphological knowledge to spelling French word endings that contain morphological information.

The present study aimed to increase our understanding of the development of morphological knowledge and assess how morphological knowledge affects spell words with morphogrammes. There were three major goals for this study. The first goal was to thoroughly assess the construct of morphological knowledge by analysing the measures used to assess it in terms of the explicit morphological awareness needed to perform each of the measures. Having examined the nature of the construct, the second goal was to replicate previous findings showing that morphological knowledge predicts the accurate orthographic representation of morphogrammes. The final goal of the study was to explore whether children use SLEs in novel orthographic situations, and if so, under what circumstances thus providing a deeper understanding of how children adapt to a challenging aspect of French orthography.

The novelty of the present study was to assess, in a sample of children from grades 1 to 3, the broader construct of morphological knowledge by using multiple measures that were assumed to differ on the degree of explicit reasoning their required. Although some studies have used multiple concurrent assessments to quantify morphological knowledge (e.g., Casalis and Louis-Alexandre, 2000; Duncan et al., 2009), these studies only describe grade effects within each measure and do not report differences in grade performance across measures. The present study used four measures: a relational assessment task, two decomposition tasks and an analogy task. This array of measures, which require varying levels of implicit and explicit morphological reasoning, was thought to be particularly suitable for acquiring a comprehensive estimate of elementary children’s morphological knowledge. Thus, even if young children struggle to perform highly explicit morphological tasks, such as analogy, they might still display their morphological skill in implicit tasks, such as relational judgements. It was hypothesized that task difficulty would increase as the amount of explicit morphological manipulation needed to solve said task increased. In other words, relatedness was hypothesized to be easier than decomposition, which was in turn hypothesized to be easier than analogy. The rank order of task difficulty was not expected to change across grades. Furthermore, although these four tasks were hypothesized to vary in the levels of implicit and explicit cognitive processing required, they were still considered to be part of one unifying construct, and that under factor analysis, they would load onto a single factor. Before discussing further goals of this study, however, it would be prudent to first address a controversy with the use of decomposition tasks.

Although widely used (Tyler and Nagy, 1989; Carlisle, 2000; Casalis and Louis-Alexandre, 2000; Fejzo, 2016), decomposition tasks have been criticized due to ambiguity as to whether children use morphological knowledge, phonological decoding or general vocabulary knowledge to complete them. Generally, the phonology of a root word is preserved to some degree within its derivative form – thus, we cannot know if a child successfully segments a word because they recognize the semantic relation between the root and derivative, or because they hear a “smaller word” within the derivative, or both. To address this, a novel decomposition task was developed. Unlike traditional decomposition tasks, which note only if the child identified the semantic root of a word, this new task also tracked if the child identified a phonologically “smaller” word – for example, the word *lire* [read] in *faiblir* /fɛbliʁ/ [weaken]. As an additional measure of phonological strategy use, some items of this task were unaffixed, meaning a smaller root could not be identified – for example, the word *pardon* /paʁdɔ̃/ [pardon]. If a child identified what they believed was a smaller word within this item – for example, *don* /dõ/ [donation] – then they must have used a phonological strategy to do so. By accounting for children’s proclivity to use phonological strategies to decompose words, it may be possible to partial out this variability, creating a more accurate measure of children’s morphological reasoning. The efficacy of the new morphological-phonological decomposition task was assessed and compared to a more “traditional” decomposition task. While in general the morphological-phonological decomposition should be no more difficult than the traditional decomposition task, the items for which children must recognize that a semantically smaller word cannot be identified could prove more difficult, and thus, it was hypothesized that this task would be more difficult than the traditional decomposition task, though not so difficult as the analogy task.

Once the structure of morphological knowledge was established, it was possible to test whether it was statistically and significantly linked to children’s accurate orthographic representations of SLEs in words. General experience (approximated by grade level), decoding skills and general orthographic representations of words were included as covariates in the model before adding morphological knowledge. Based on prior research, morphological knowledge was expected to provide a small but significant contribution to children’s morphogramme spelling after accounting for other early literacy skills.

One final objective of this study was to examine whether children over-generalize SLEs when spelling novel words. Although the nature of spelling errors children make with morphogrammes is well documented (e.g., Sénéchal, 2000; Sénéchal et al., 2016; see also Bosse and Pacton, 2006; Quémart and Casalis, 2017), their general use of SLEs is not well understood. It is possible that, just as children go through a period of overgeneralizing morphemes (Carlisle, 2000), they may also go through a period of overgeneralizing morphogramme-like letter endings. A series of pseudowords, each with a rime that may elicit an SLE, were created to assess if children overgeneralized their use of SLEs. Pseudowords ensured that children’s prior knowledge did not confound their spelling. If children did overgeneralize SLEs, it was expected that the letters *t* and *e* would be the most common, being the most and second most frequent SLEs, respectively (Gingras and Sénéchal, 2017). However, children in grade 1 were not expected to overgeneralize SLEs, as it seems unlikely that they would have sufficient experience with written language to begin forming and over using these sorts of schemas.

## Methods

### Participants

One hundred and twenty-nine children between 5 and 8 years old were recruited from four francophone schools in Gatineau, Canada, in 2007. Three children withdrew from the study partway through testing, and an additional three children were omitted due to substantially incomplete data – the final sample size was 123. Children were recruited directly through the schools – a consent form was handed out by teachers to the children and signed by parents at home. Forty-six children were in grade 1 (*M =* 6.3 years, SD = 3.6 months; 25 boys), 51 in grade 2 (*M =* 7.4 years, SD = 4.1 months; 33 boys) and 26 in grade 3 (*M =* 8.5 years, SD = 3.5 months; 10 boys). All children spoke French, although many children spoke or were exposed to other languages in the home, including English (70.5%), Arabic (7.7%), Somali (3.1%), Greek (1.6%), Italian (1.6%), German (<1%), Russian (<1%), Creole (<1%), Spanish (<1%) and Berber (<1%).

## Materials

### Children Reading Frequency

Parents reported on children’s frequency weekly readings at bedtime and other times on a nine-point scale ranging from never to reading more than seven times a week. Parents also reported on the frequency with which their child read in French on a four-point Likert scale ranging from *never* = 0 to *very often* = 3.

### Morphological Knowledge

Multiple morphological assessments were used to gain a comprehensive estimate of children’s implicit and explicit morphological reasoning and consequently achieve a well-rounded estimate of morphological knowledge. The four tasks assessed children’s understanding of the relationship between roots and derivatives, their ability to deconstruct multimorphemic words, the difference between children’s phonological and morphological decoding of words and their ability to derive words through analogy. In all tasks, practice items with feedback were provided at the beginning to familiarize the child with the task; no feedback was provided for test items. Each task is described next, and task items are in Appendix. The internal consistency measure Cronbach’s *α* was used to assess inter-reliability for all measures in this study. Generally, Cronbach’s *α* is considered acceptable if it falls between 0.70 and 0.95 – any lower suggests that the scale has substantial measurement error, and any higher suggests redundancy between the items (Cortina, 1993; Tavakol and Dennick, 2011).

#### Relatedness

This spoken test was developed by Colé et al. (2004) and assessed children’s ability to recognize whether two words were morphologically related. The task included 20-word pairs each consisting of a root word and a derived word, and all derivatives were suffixed. The suffixes (or pseudosuffixes) were phonologically transparent – this eased the difficulty of the task, as children find derivatives without phonological shifts easier to decompose than those with phonological shifts (Carlisle, 2003). Within each pair, one word was presented as a potential root and the other as a potential derivative. Ten pairs of words were morphologically related – for example, *amour* [love] and *amoureux* [amorous]; the remaining 10 pairs were orthographically and phonologically similar but did not share a sematic root – for example, *heure* [hour] and *heureux* [happy]. The assistant administering the task told the child that they would hear two words that sounded similar, and that the child should say whether the words were part of the same “family” or not. Children received one point for each correct answer. For this measure, inter-item reliability was acceptable (Cronbach’s *α* = 0.69).

#### Decomposition

Children were orally presented 20 multimorphemic words (19 two-morpheme and 1 three-morpheme words) and asked to find the “smaller word”, or root word, within each item – for example, a smaller word within *oreiller* [pillow] is *oreille* [ear]. All items were suffixed derivatives. Children received one point for each word they successfully segmented into its root form. Although it was also desired to analyse children’s phonological decompositions – that is, when children identified a smaller word *via* the sound of the derived word, and not through analysing semantic relations – the archival nature of the data did not allow this. Inter-item reliability was good (Cronbach’s *α* = 0.83).

#### Morphological-Phonological Decomposition (Morpho-Phono Decomposition)

Children were told that they would be presented with a series of words, some of which may contain a smaller word. The children were asked to identify if a smaller word existed, and if so, what it was. The task was developed for the present study and included 10 items that could be morphologically decomposed (with an approximately equal number of prefixed and suffixed words), and 10 items that were unaffixed (i.e., had no smaller root within them). Unlike the previous test, where children were assessed solely on their ability to identify the morphological root, this test assessed whether children provided a morphological decomposition of a word (for example, identifying *bond* [jump] within *rebond* [rebound]) or a phonological decomposition (*lire* within *faiblir*). Children’s answers were scored as either morphological, phonological or other: a morphological answer indicated the child correctly identified the root of an affixed target word or correctly surmised that an unaffixed target word could not be morphologically decomposed; a phonological answer indicated the child used phonological decomposition to *incorrectly* identify a word other than the root of an affixed target or to identify a smaller word in an unaffixed target; other answers included non-responses, reporting only the first letter of the target word or saying there was no root in a word that could be morphologically decomposed. Inter-item reliability of the scale as a whole was poor (Cronbach’s *α* = 0.64) with affixed items (Cronbach’s *α* = 0.44) having lower reliability than unaffixed items (Cronbach’s *α* = 0.53). Cronbach’s *α* operates under the assumption of tau equivalence (Cortina, 1993) – that is, it assumes that item standard deviations are equivalent – and it underestimates reliability when this assumption is violated. Item analysis revealed substantial differences between item standard deviations, with the lowest being SD = 0.25 for the item *droitier* [right-handed person] and highest being SD = 0.83 for the item *faiblir* [weaken]. However, no appreciable increase in reliability was observed when removing individual items from the scale. Consequently, the scale was left whole for future analyses.

#### Analogy

This task was adapted from Sénéchal (2000) and designed to assess children’s ability to form derivatives from root words using analogy. Children were orally presented two words that shared a morphological relation and were next provided the first item of a second pair. The children were then asked to deduce the missing item. For example, given the sequence “*gris* [grey (masc.)]: *grise* [grey (feminine form)] ∷ *blond* [blond (masc.)]: _______”, the child would be expected to derive *blonde* [blond (feminine form)]. The morphological transformation was always suffixation. There were 20 items, and children were scored based on the number of items derived correctly. Inter-item reliability was good (Cronbach’s *α* = 0.83).

### Representing Words With Silent-Letter Endings

The SLE orthographic recognition task was a written, classroom administered task assessed children’s representation of silent-letter endings similar to the Orthographic Coding task developed by Olson (for a critical review, see Olson et al., 1994). Although this task involved a reading component, children had to access the orthographic representation of the silent-letter ending to answer correctly. Specifically, children were provided 30 sets of morphogramme words and asked to choose the correctly spelled word from three alternatives, all with an identical pronunciation. These alternatives included the word spelled with the correct SLE (e.g., *chocolat* [chocolate]), the word spelled with an incorrect letter ending (e.g., *chocolas) and the word with the silent letter omitted (e.g., *chocola). Children answered the items at their own pace and received one point for each correctly identified word. Inter-item reliability was acceptable (Cronbach’s *α* = 0.70). The items are in the Appendix.

### General Orthographic Representations

The general orthographic recognition task, a classroom administered task, provided a more general view of children’s orthographic representation. It is similar to Olson’s Orthographic Coding task (for a critical review, see Olson et al., 1994). Children were presented with 30 pairs of words with an identical pronunciation but alternate spellings – for example, *jambe* (leg) and *jembe. To answer correctly, children had to access the accurate orthographic representation of the ambiguously spelled phoneme. Children received one point for each correct answer. Inter-item reliability was acceptable (Cronbach’s *α* = 0.72). The items are in the Appendix.

### Phonological Decoding

This written, classroom administered task presented children with 30 pairs of non-words (e.g., *fraze* and *traze*), one of which was phonetically identical to a real word (e.g., *phrase* [sentence]). The children were asked to identify which non-word was pronounced like a real word. They received one point for each correct answer. Inter-item reliability was poor (Cronbach’s *α* = 0.63). However, there were only modest differences between item standard deviations – the lowest was SD = 0.12 for the item *harmé* and the highest was 0.50 for the items *gam* and *eguiye*. There were no appreciable increases in reliability when removing individual items from the scale. As with the morpho-phono decomposition task, it was decided to keep the scale whole for future analyses. The items are in the Appendix.

### Over-Extension of Silent Letters

The Silex database of French orthography (Gingras and Sénéchal, 2017) reports *e*, *t*, *d*, *s* and *x* to be the five most frequent silent-letter endings overall. However, the occurrence of any particular morphogramme is strongly conditional on the preceding phonological context. For example, many French words whose final syllable sounds as /aʀ/ end in a silent “*d*”, as in *renard* [fox] and *canard* [duck]. Furthermore, children in grades 1–3 are sensitive to this context (Sénéchal et al., 2016). Eighteen two-syllable pseudowords, each designed to elicit a SLE, were developed to assess whether children would over-extend their use of SLEs. To elicit a breadth of SLEs, six different phonological endings were used: three oral vowel endings (/o/, /i/ and /a/), two /ʀ/ endings (/oʀ/ and /aʀ/) and one nasal vowel ending (/ã/). The task items, in order of presentation, were juti, fenar, pada, falo, renan, cajor, rajo, bivar, mouco, ciror, moufa, bonan, juna, cabi, ravor, cinan, mofi and dassar.

This task was administered by classroom and presented as a spelling task. Children were provided a sheet of paper on which to write their answers. Given that only the spelling of the words’ endings was of interest, it was decided to ease the difficulty of the task by providing the children with the first syllable of the word and asking them to write the second syllable. The research assistant administering the task first explained that the children would be attempting to spell some “made up” words, and that children should try and provide an answer even if they are unsure of the proper spelling. The assistant dictated each pseudoword twice. An experimenter later totalled which of the five target SLEs, and how many, children used in their spelling. Inter-item reliability was good (Cronbach’s *α* = 0.86).

### Procedure

The assessments were completed in school, during regular hours and at the teacher’s convenience. All tasks were administered by a trained research assistant. After a brief period of time to acclimatize the students to the presence of the research assistant, testing began. Each child participated in a classroom wide session, during which the test for over-extension of silent letters, the SLE orthographic recognition, the general orthographic recognition and the phonological decoding tasks were administered in this fixed order – the session in its entirety took 30–40 min. Children were tested in early winter, and there was concern that grade 1 children would not yet have enough experience with reading on their own to complete the aforementioned tasks. It was decided to exempt grade 1 children from the decoding and the orthographic recognition tasks to exclude potential confounds with their reading ability. Children from all grades each participated in a one-on-one session with the research assistant, during which the four morphological knowledge assessments were administered in the following order: relatedness, decomposition, analogy and morpho-phono decomposition. The one-on-one session took place in a quiet space at the school, such as an empty classroom or library. These sessions took 10–15 min.

## Results

### Preliminary Analyses

Preliminary analyses were conducted to assess the effects of missing data, outliers and child language. Because no variable was missing more than 5% of its data, missing data were imputed using multiple hot-deck imputation (Crammer et al., 2016). For each measure with missing data, potential information donors were identified by grade, gender and the remaining items in the measure. Given that missing data were minimal, the potential variance lost using this “simple” imputation method was trivial (Cranmer and Gill, 2013). Examination of distributions revealed the presence of minor outliers in the decomposition task, the morpho-phono decomposition task, the analogy task and the SLE orthographic recognition task. Most outliers represented a score that was low for the sample. However, given that the deviation from the expected range of scores was fairly minimal, with outliers often being only one or two points below the lowest expected score, it was decided that they would not pose a significant threat to the distribution of the data, and they were left unchanged. Finally, given the high percentage of multilingual children in the sample, correlations were drawn between all measures and children’s status as a French monolingual or multilingual student to identify potential differences between these groups. All correlations were non-significant (largest correlation was *r* = 0.14, *p* = 0.13); thus, child language is not discussed further.

### Morphological Knowledge

#### Morphological Versus Phonological Strategies During Decomposition

The morpho-phono decomposition task was designed to provide an insight into children’s use of phonological rather than morphological strategies when decomposing words. As such, the distribution of answer types was analysed with two goals in mind – to assess if the morpho-phono task did indeed capture children’s use of phonological answers, and if so, how best to account for them in future analyses. Recall that morphological answers were those wherein the child successfully identified the root of an affixed word, or correctly surmised that an unaffixed word had no internal root (i.e., it is, in and of itself, the root). Phonological answers were those wherein a child phonologically decomposed a word to identify the “smaller word”. Other answers included no responses, child answered with a non-word or child said that they did not know. The mean number of morphological, phonological and other responses is presented in Table 1. All values are significantly greater than zero (*t*s > 3.39, *p* < 0.0027), indicating that at all grades, children produced a significant number of morphological, phonological and other responses.

**Table 1:** Mean responses (and standard deviation) on the morpho-phono decomposition task as a function of response type and grade.

	Affixed words (max. 10)			Unaffixed words (max. 10)		
	Grade 1	Grade 2	Grade 3	Grade 1	Grade 2	Grade 3
Morphological	4.8 (2.2)	6.2 (2.1)	7.3 (1.6)	2.4 (1.6)	3.0 (1.7)	3.7 (1.7)
Phonological	1.2 (1.0)	0.8 (1.0)	0.6 (0.9)	4.6 (1.6)	5.5 (1.6)	5.0 (1.7)
Other	4.0 (2.1)	3.0 (2.0)	2.0 (1.3)	3.6 (1.7)	2.0 (2.0)	1.7 (0.9)

*Morphological answers were the (1) successful identification of the root of affixed words or (2) correct judgement that unaffixed words could not be decomposed. Phonological answers were the (1) inaccurate identification of a smaller word other than the root in affixed words or (2) inaccurate identification of a smaller word in unaffixed words*.

Examination of morphological strategy use in Table 1 revealed that children found decomposing affixed words easier than stating that an unaffixed word could not be decomposed. The examination of phonological strategies revealed a different pattern altogether. Children seldom used a phonological strategy for affixed words, whereas they often decomposed unaffixed words. Furthermore, this pattern of response generally held constant for all affixed and unaffixed items – that is to say, no one affixed or unaffixed item received substantially higher quantities of morphological or phonological decompositions.

The contrast between strategy use for affixed and unaffixed words is instructive because it suggests that, in some circumstances, children’s performance in decomposition tasks might be influenced by their propensity to use phonology to decompose words. Given this propensity and the novelty of the task, we examined whether to adjust children’s scores for subsequent correlational analyses. To do so, we examined the pattern of correlations on this task. We found that children’s morphological responses on the affixed items were not associated with those on unaffixed items (*r* = 0.08 after controlling for grade). Given this pattern, it is children’s morphological responses on affixed items that were used in the subsequent correlational analyses. We also conducted an additional verification as to whether adjusting these scores by subtracting the number of phonological responses from the morphological responses to affixed items would alter findings, and it did not. Consequently, the unadjusted morphological responses on the affixed items were used in the subsequent analyses.

#### Growth in Morphological Knowledge

Descriptive statistics for the four morphological knowledge tasks are found in Table 2. Performance on the morphological relatedness task was significantly better than the expected 50% chance of success for all grades [smallest was *t*_Grade 1_(45) = 6.55, *p* < 0.001], although children in grade 1 did not perform above chance for unrelated word pairs (*t* [45] = −0.41, *p* = 0.69).

**Table 2:** Mean performance (and standard deviation) on morphological tasks as a function of grade.

	Grade 1	Grade 2	Grade 3
	*n* = 46	*n* = 51	*n* = 26
Relatedness (20)	12.57 (2.66)	14.98 (2.60)	16.19 (2.59)
Related word pairs (10)	7.74 (1.87)	8.06 (1.21)	8.30 (1.22)
Unrelated word pairs (10)	4.83 (2.91)	6.92 (2.37)	7.92 (2.19)
Decomposition (20)	11.72 (3.96)	15.00 (2.40)	17.35 (1.62)
Morpho-phono decomposition (20)^a^	7.54 (2.86)	9.82 (3.05)	11.65 (2.38)
Analogy (20)	2.94 (2.09)	6.37 (2.73)	7.96 (2.78)

*The maximum score is presented in parentheses following each measure*.

a *For mean correct responses on the affixed and unaffixed items, see morphological responses in Table 1*.

**Figure 1:**
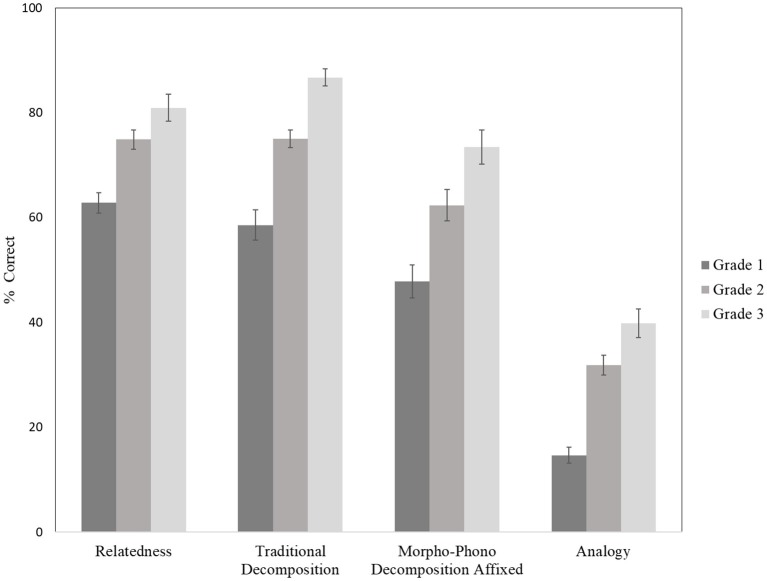
Mean per cent correct (and SEMs) on four morphological tasks as a function of grade.

A MANOVA analysis on the four morphological tasks revealed a significant main effect of grade (Pillai’s = 0.521, *p* < 0.001). This was followed by a 3 (Grade: 1 vs. 2 vs. 3) × 4 (Task: relatedness vs. decomposition vs. morpho-phono decomposition vs. analogy) mixed-design ANOVA, with grade as the between-subjects factor and task as the within-subjects factor. These analyses were conducted to test the hypothesis that tasks that can be solved through implicit morphological processing are easier than those requiring explicit morphological awareness. The use of a repeated-measures ANOVA to assess mean differences among morphological tasks was justified by conceptualizing the tasks as four different treatment levels varying in explicit cognitive requirements. The task order, from easiest to hardest, was hypothesized to be relatedness, decomposition, morpho-phono decomposition and analogy. The analysis revealed a significant main effect of grade, *F*(2, 120) = 49.58, MSE = 0.01, *p* < 0.001, and task, *F*(3, 360) = 281.79, MSE = 0.02, *p* < 0.001, but the interaction was not significant, *F*(6, 360) = 1.01, MSE = 0.02, *p* = 0.42. Figure 1 illustrates the similarity across grades in task difficulty. Repeated contrasts indicated that, as expected, performance improved from grades 1 to 2, *F* = 51.71, *p* < 0.001, and from grades 2 to 3, *F* = 13.54, *p* < 0.001. Within-subjects contrasts revealed no mean difference in children’s performance on the relatedness and traditional decomposition tasks, *F*(1, 120) < 1, ns; however, morpho-phono decomposition was harder than the traditional decomposition, *F*(1, 120) = 38.78, MSE = 0.04, *p* < 0.001, and analogy was harder than morpho-phono decomposition, *F*(1, 120) = 255.51, MSE = 0.05, *p* < 0.001. All significant contrasts exceeded the Bonferroni correction of *p* < 0.008. In general, this pattern of results provides support for the hypothesis that tasks that rely on explicit morphological awareness are more difficult than those that can be solved using implicit processing.

#### The Structure of Morphological Knowledge

The correlations among the morphological measures ranged from 0.43 to 0.53. These coefficients were significant, positive and moderate in strength, indicating that children who scored high on any one measure of morphological knowledge tended to score high on others as well. An exploratory factor analysis was conducted to assess the hypothesized factor structure representing morphological knowledge. The classic eigenvalues greater than one criterion were used to determine the number of factors to retain (Matsunaga, 2015). The first eigenvalue at 2.45 was the only eigenvalue to exceed one. It accounted for 61% of the observed variance – thus, a one factor solution was considered appropriate. Factor loadings, ranging from 0.64 to 0.73, and communalities, ranging from 0.42 to 0.57, were reasonably strong. The measures are well represented in this factor space, and no one measure accounted for a substantially larger amount of variance. This confirms the hypothesis that, despite the measures assessing different levels of implicit or explicit morphological reasoning, they all assess a unified construct. The factor scores from this analysis were used as the measures of morphological knowledge in all subsequent analyses.

### Reading Behaviours at Home and Literacy Skills

As shown in Table 3, parents reported, on average, that children read in French very often and read frequently on a daily basis. As shown in the table, there was little variation across grades in the children’s experiences. Indeed, grade was not a significant factor in a MANOVA that included the three questions.

**Table 3:** Mean performance (and standard deviation) on reading behaviours and literacy skills.

	Grade 1	Grade 2	Grade 3
**Reading at home**			
Reading at bedtime^a^	4.87 (2.25)	4.43 (2.53)	3.72 (2.23)
Reading at other times^a^	4.45 (2.04)	3.85 (2.61)	4.09 (2.75)
Child reads in French^b^	1.98 (0.80)	2.02 (0.77)	1.76 (0.97)
**Literacy skills** ^c^			
Decoding	–	23.43 (3.60)	23.46 (3.20)
General orthographic rec.	–	20.88 (3.87)	24.12 (3.58)
SLE orthographic rec.	–	16.59 (4.81)	20.88 (3.87)

*Literacy tasks were not administered in Grade 1. rec. = recognition; SLE = silent-letter ending*;

a *0 = never; 1 = once a week; 2 = twice a week; …; 7 = seven times a week; 8 = more than seven times a week*;

b *0 = never; 1 = rarely; 2 = often; 3 = very often*;

c *There are 30 items in each literacy task*.

The descriptive statistics for the literacy measures are also shown in Table 3. Mean performance on phonological decoding, general orthographic recognition and SLE orthographic recognition each exceeded their respective chance performance levels (*ts*_Grade 2_ = 16.74, 10.85, 9.78, *p*s < 0.001). Children in both grades performed equally well on the decoding task. Children’s performance on the two orthographic recognition tasks was analysed with a 2 (grade: 2 vs. 3) × 2 (task: general vs. SLE orthographic recognition) mixed-design ANOVA. The analysis revealed a significant main effect of grade, *F* (1, 75) = 18.03, MSE = 29.34, *p* < 0.001, and task, *F* (3, 360) = 65.93, MSE = 510.22, *p* < 0.001, but the interaction was not significant, *p* = 0.13. Although children’s performance improved across grades, children in both grades had more difficulty identifying the correct spelling of words ending with a silent letter (SLEs) as compared to the correct spelling of words in general (i.e., without SLEs).

### Morphological Knowledge and Representing Silent Letters

In order to assess the central hypothesis that morphological knowledge is positively and robustly associated with children’s ability to represent silent letters correctly, it was necessary to also examine their relations with other measures. As shown in Table 4, representing silent letters accurately and morphological knowledge were significantly correlated, and both were also associated with general orthographic recognition, the frequency of children’s independent reading and grade level. General orthographic recognition was positively associated with reading behaviours and grade. Children’s decoding skills, however, were not associated with any measure.

**Table 4:** Pearson correlations among key variables for children in grades 2 and 3.

	1	2	3	4	5	6
1. SLE orthographic rec.	–					
2. Morph. knowledge	0.439^**^	–				
3. General orthographic rec.	0.696^**^	0.365^**^	–			
4. Decoding	0.149	0.237	0.049	–		
5. Reading frequency	0.244^*^	0.250^*^	0.352^**^	0.091	–	
6. Reads in French	0.154	0.224^*^	0.355^**^	0.127	0.727^**^	–
7. Grade	0.427^**^	0.406^**^	0.379^**^	0.004	−0.051	−0.149

*SLE, Silent-letter endings; orthogr. rec., orthographic recognition; Morph., morphological*.

* *p < 0.05*;

** *p < 0.01*.

A fixed-order regression analysis was used to test whether morphological knowledge remained associated with children’s representation of SLEs after controlling for key predictors. The order of entry of the control variables was determined according to their theoretical proximity to SLE representation, from the farthest removed to the closest. As shown in Table 5, grade level accounted for a significant 18% of variance, the two reading frequency measures added 7%, while general orthographic recognition contributed 26.7% more variance. As predicted, morphological knowledge entered last in the equation accounted for 2.5% unique variance in children’s ability to recognize SLEs correctly.

**Table 5:** Fixed-order hierarchical regression for the orthographic recognition of silent-letter endings.

Variable order	*R*^2^	∆*R*^2^	*F* change	Final			95% CIs	
				*B*	SE	*β*^a^	Lower	Upper
1. Grade	0.182	0.182	16.69^***^	1.01	1.057	0.101	−1.017	3.98
2. Reading frequency	0.255	0.073	3.58^*^	0.128	0.138	0.109	−0.147	0.403
3. Reads in French				−1.025	0.755	−0.166	−2.531	0.482
4. General ortho. rec.	0.524	0.269	40.73^***^	0.774	0.124	0.611	0.528	1.021
5. Morph. knowledge	0.550	0.025	3.99^*^	1.397	0.699	0.186	0.004	2.790

*Ortho. rec., orthographic recognition; Morph., morphological*.

* *p = 0.05*;

*** *p = 0.001*;

a *Final β s are statistically significant when the CI range excludes zero*.

### Do Young Children Overgeneralize the Spelling of Silent-Letter Endings?

Included in this study was a dictation of pseudowords with endings that are often followed by as silent consonant in French. To assess whether children overgeneralized their use of SLEs, their proportional use of SLEs following pseudowords with various rimes was tabulated – the results are presented in Table 6. Overall, children rarely use SLEs when spelling unfamiliar words, although their proportion of usage does increase slightly with age – only 8% of grade 1 children’s answers included any kind of SLE, while 21% of grade 3 children’s answers included an SLE. Furthermore, there was some variability based on the phonological rime – while between 1 and 5% of children provided an SLE following the rime /o/, 17–41% provided an SLE following /aʀ/. When SLEs were used, the letter *e* was most common across all ages and phonological contexts, though particularly following the oral vowel /i/ and words ending in an /ʀ/. Strikingly, children in grades 2 and 3 seemed to have some sensitivity to the letter “d” in specific contexts. Specifically, they used a terminal *d* following /aʀ/, as in *fenar,* or /oʀ/, as in *travor*. Furthermore, within /aʀ/ words, there existed a proportional decrease in the use of *e* over *d* as children entered grade 3, as a *χ*^2^ test of homogeneity found that the distribution of SLE responses within this rime was not equivalent across grades [*χ*^2^ (4) = 39.4, *p* < 0.001]. This provides some evidence that, by this age, children restrict the context in which they use silent-letter endings to the most appropriate letter. However, children’s use of the silent letters *t*, *s* and *x* is almost non-existent.

**Table 6:** Proportion of children’s silent-letter endings during pseudoword dictation as a function of phonological rime and grade.

Rime	Grade	*e*	*t*	*d*	*s*	*x*	Omission
/a/	1	0.01	–	–	–	–	0.99
	2	0.03	0.01	–	–	–	0.96
	3	0.03	–	0.01	–	0.01	0.95
						
/i/	1	0.04	–	–	0.01	–	0.96
	2	0.10	–	–	–	–	0.90
	3	0.24	0.01	–	–	–	0.74
						
/o/	1	0.01	–	–	–	–	0.99
	2	0.01	–	–	–	–	0.99
	3	0.03	–	0.01	0.01	–	0.95
						
/oʀ/	1	0.21	–	–	–	–	0.79
	2	0.30	–	0.05	–	–	0.65
	3	0.18	0.05	0.13	–	0.01	0.63
						
/aʀ/	1	0.17	–	–	–	–	0.83
	2	0.16	0.01	0.10	–	–	0.72
	3	0.06	0.06	0.28	–	–	0.59
						
/ã/	1	0.04	–	–	–	–	0.96
	2	0.05	0.02	–	0.01	–	0.93
	3	0.03	0.04	0.03	–	0.01	0.90
Average	1	0.08	–	–	–	–	0.92
	2	0.11	0.01	0.03	–	–	0.86
	3	0.09	0.03	0.08	–	0.01	0.79

## Discussion

The present study documented the growth of children’s morphological knowledge across grades 1–3. Although children’s performance on four morphological knowledge tasks improved across grades, tasks that required more explicit morphological processing were harder than those relying on implicit knowledge. Yet, all tasks loaded on a single morphological knowledge factor and this factor explained additional unique variance in children’s accurate spelling of morphogrammes – silent-letter endings (SLEs) that are pronounced in derived members of a word family. Finally, a pseudoword spelling task revealed some evidence that, with experience, the phonological rime might prime the use of SLEs. Each of these findings is discussed in turn.

### Morphological Knowledge

Prior research suggests that children’s capacity for explicit morphological manipulation is unstable in young elementary students, particularly among first graders (Casalis and Louis-Alexandre, 2000; Kuo and Anderson, 2006). Several studies have used multiple morphological measures to gain a more complete understanding of children capabilities (e.g., Casalis and Louis-Alexandre, 2000; Berninger et al., 2009; Duncan et al., 2009; Desrochers et al., 2018); however, these studies only report between-grade differences in children’s performance on disparate measures. Without examining within-grade differences, it remains unclear how measure selection influences the structure of the final morphological construct. In the present study, four tasks were chosen – relatedness, traditional decomposition, morpho-phono decomposition and analogy – each assumed to require progressively more explicit morphological manipulation. In general, tasks believed to require more explicit cognitive effort were more difficult – analogy was significantly harder than morpho-phono decomposition, which was in turn more difficult than traditional decomposition and relatedness.

Examination of the mean scores of each grade across tasks illustrated the importance of choosing appropriate measures for a particular age range. Although Sénéchal (2000) and Desrochers et al. (2018) report that analogy tasks are appropriate for children beginning grade 2, in this study the grade 1 children tested in the middle of the school year struggled markedly, answering on average just two of 20 questions correctly. By contrast, children in grade 3 performed near ceiling level on the more implicit relatedness and traditional decomposition tasks. In other words, tasks requiring marked explicit manipulation appeared to be almost too difficult for first graders, but tasks which required very little appeared too easy for third graders. Clearly, due to the wide variation in skill level across this population, no one task registered a suitable breadth of variance – only by using multiple measures targeting different skill levels was it possible to gain a comprehensive view of morphological knowledge across these three grades. That said, and in spite of the range of difficulties presented by these measures, they all loaded on a single factor, indicating they assessed a unified construct. Ours is not the first factor analysis performed to assess the dimensionality of morphological knowledge – in a sample of fourth graders, Spencer et al. (2015) found that a one factor solution performed adequately in comparison to two-factor solutions based on oral versus written measure administration and oral versus written child response; by contrast, Tighe and Schatschneider (2015) confirmed a two-factor solution composed of real-word tasks and pseudoword tasks in a sample of adult basic education students. To our knowledge, however, no other factor analysis has been performed to assess the structure of morphological knowledge on the dimensions of implicit versus explicit reasoning, nor has one been performed in so young a cohort. Unfortunately, due to limited sample size, we were unable to examine whether the factor structure of morphological knowledge changes across age groups. Given how rapidly children’s morphological reasoning evolves after beginning school (Anglin et al., 1993; Carlisle and Nomanbhoy, 1993; Casalis and Louis-Alexandre, 2000; Duncan et al., 2009), the morphological structure of elementary school children may differ from middle to high school children – for example, perhaps a two-factor solution presents in older children once sufficient experience in explicit morphological reasoning is achieved. Future research may wish to examine how the structure of morphological knowledge evolves with children’s exposure to reading and writing in school.

Theoretical concerns have been voiced that traditional decomposition tasks can be solved with phonological strategies rather than morphological ones, inflating children’s apparent morphological skill. The morpho-phono decomposition task was developed to explore this possibility by first using items that may elicit phonological decompositions, and noting when children use an alternate decomposition strategy. At all grades, children were observed to make a significant number of phonological decompositions when presented with unaffixed words, despite accurately decomposing affixed words. There are at least two interpretations of this finding. First, young elementary-school children may indeed use a phonological strategy when decomposing words, and this suggests the need to adjust children’s performance accordingly as well as including other types of morphological measures as was done in the present study. Second, it is possible that the instructions of finding a smaller word might have biased children answers. Studies of child response bias have determined that children are often reluctant to say that they do not know the answer (Earhart et al., 2014), and that this bias is exasperated in closed-ending type questions (Waterman et al., 2001). One study found that, despite high accuracy in identifying nonsensical or unanswerable questions, children still attempted to answer more than 70% of nonsense question (Waterman et al., 2010). It is possible that children, being primed to look for smaller words (i.e., roots) and reluctant to provide no answer, instead utilized phonological decomposition to achieve a response. Nevertheless, the morpho-phono decomposition task provided a method of quantifying children’s proclivity for phonological decomposition, by recording children’s use of phonological decomposition when answering affixed words, as opposed to traditional decomposition tasks, which merely report whether children provide the correct answer. Furthermore, acquiring this information about children’s phonological decomposition came at a paltry increase in time and effort on the part of the researchers, making the morpho-phono decomposition task an economical way to quantify both children’s morphological knowledge and their reliance on phonology. Although correcting or not for children’s phonological decomposition on the affixed words yielded identical findings in the present study, future research is needed to understand better why children are likely to use phonological strategies on unaffixed words.

### Orthographic Representations of Morphogrammes

Having exhaustively examined the morphological construct, the next goal of the present study was to add to a small body of research assessing the contributions of morphological knowledge to spelling in general (for reviews, see Sénéchal and Kearnan, 2007; Pacton and Deacon, 2008; Abbott et al., 2016), as well as morphogramme spelling in particular (Casalis et al., 2011). Indeed, after controlling for grade, print exposure and general orthographic representations, morphological knowledge explained an additional 3% unique variance in morphogramme recognition, a result comparable to the 2% reported in the study of Sénéchal (2000) and the 4% reported in the study of Fejzo (2016). Furthermore, unlike in the study of Sénéchal (2000), our analogy to print exposure – reading at home – was a significant predictor of morphogramme recognition (when entered after grade), and yet morphological knowledge continued to explain additional variance. Print exposure predicates orthographic redundancy, as greater exposure provides more opportunities for a child to encode members of the same word family (Ouellette, 2010; Conrad et al., 2013). The fact that morphological knowledge predicted morphogramme recognition in spite of this indicates that the advantage morphological knowledge provides to the orthographic representation of SLEs goes beyond the benefits provided by orthographic redundancy in large-word families. This adds to a body of research showing that morphological knowledge provides a unique benefit to children’s early spelling, presumably by bolstering the recollection of phonologically underspecified letters and, therefore, fostering the formation of complete orthographic representations. Importantly, the benefits afforded by morphological knowledge cannot be readily explained by the orthographic redundancy between roots and their silent-letter revealing derivatives (Pacton et al., 2018). Notwithstanding the beneficial effects of morphological knowledge, recent evidence has also shown learning effects due to the frequency of occurrence of the silent-letter themselves (Sénéchal et al., 2016; Gingras and Sénéchal, 2019). As such, children seem to harness multiple learning mechanisms when acquiring their orthographic lexicon.

### Overgeneralization of Silent-Letter Endings

Silent-letter endings present great difficulty to children’s spelling, with children omitting the SLE in 52–66% of instances (Sénéchal, 2000; Sénéchal et al., 2016). In the present study, children of all grades rarely used SLEs when spelling to dictation the rime of pseudowords containing terminal phonemes warranting SLEs. While first graders, being novice readers, were not expected to have the experience necessary to understand the important of SLEs in French orthography, it was expected that older children would use more SLEs when writing pseudowords, given that they have more established representations of these letters (Gingras and Sénéchal, 2019). However, there was a small trend for older children to use more SLEs than younger ones. It is possible that, given the long period it takes for children to master spelling SLEs (Québec Ministère de l’Éducation et de l’Enseignement supèrieur, 2009; Gingras and Sénéchal, 2019), the expected overgeneralization does not occur until a later age.

The five most common SLEs in French are *t*, *e*, *s*, *x* and *d* (Gingras and Sénéchal, 2017) In the present study, the most common letter used by children was *e*. Though it is possible that sheer frequency influenced *e*’s prevalence, the most common SLE overall, *t*, was almost never used. Furthermore, the letter *d* is the least frequent SLE among the five represented in this study – however, it was the second most used by this cohort of children. Thus, the frequency of the SLE in French orthography does not seem to drive children’s acquisition of the SLE. The use of *e* could tentatively be explained through its involvement with the French process of feminization. All nouns in French possess a grammatical gender, and many have male and female variants, which change their orthography and phonology (Jaffré and Fayol, 2006). A terminal letter *e* often denotes a feminine form of a word (e.g., *bavard* (masc.) vs. *bavarde* (fem.) [chatterbox]). Children recognize grammatical gender from infancy (Van Heugten and Shi, 2009), and the process of feminization is explicitly taught in early grades in Québec schools (Québec Ministère de l’Éducation et de l’Enseignement supèrieur, 2009). It is possible, then, that the association of *e* to this common and explicitly taught inflectional process makes it particularly salient – thus, *e* may become a “go to” SLE for novel words.

By contrast, the letter *d* is not a common SLE in French orthography, although it occurs regularly in words ending with /ʀ/ (Gingras and Sénéchal, 2017). In the present study, children used the SLE *d* almost exclusively following the rimes /aʀ/ and /oʀ/. Anecdotal evidence suggested a grade effect – in grade 1, 17% of SLE responses to the /aʀ/ rime were *e*, and 0% was *d*, but by grade 3, only 6% of SLE responses were *e*, while 28% was *d.* Perhaps the letter *d* was easier to acquire because it has a very narrow phonological domain, to which it is strongly associated. Children displayed sensitivity to the phonological context of *e* as well, using it in phonological contexts appropriate for the SLE (e.g., /i/, /oʀ/, /aʀ/) and omitting it from contexts where it occurs infrequently (e.g., /a/, /o/) or cannot occur at all (e.g., /ñ/). One avenue for future research may be to assess how the combination of frequency and phonological rime specificity affect children’s acquisition or overgeneralization of SLEs.

### Additional Limitations and Implications

Two additional overarching limitations impacted this study. The first was the study’s small and unequal sample size across grades, which impacted both the choice of statistical analyses and the generalizability of results. Many analyses, including ANOVA and regression, lose power when samples are small and uneven (Tabachnick and Fidell, 2014), although they remain fairly robust if other assumptions, such as normality and homogeneity of variance, hold true. Yet, we observed increases in performance between each grade, and the observed pattern of task difficulty across grades, shown in Figure 1, suggests more similarity than differences with increased experience. It is possible also that the smaller sample size of the grade 3 cohort means that the results of the factor analysis may be less applicable to this group, as they are underrepresented in the data. Thus, these analyses may be considered a starting point for future studies, in need of replication before findings can be confirmed.

The second overarching limitation of this study was children’s multilingualism. In this study, multilingualism did not account for differences in their early literacy skills. However, multilingualism is known to affect the development of morphological awareness (see Chen and Schwartz, 2018). It may further have an effect on the orthographic redundancy within the child’s vocabulary. For example, English was the most common second language in the present sample of children. Although English shares several morphological properties with French, such as root consistency and affixation, English seldom has SLEs. Notably, cross-language vocabulary exposure can illuminate silent letters – for example, the SLEs in *lézard* [lizard], *chocolat* [chocolate] and *confort* [comfort] are all revealed in their English equivalents. A recent study, however, showed that this beneficial effect is temporary. Jubenville et al. (2014) found, in a sample of monolingual and bilingual children schooled in French, different effects during oral vocabulary learning of the incidental presence of the printed word-to-be learned. In this study, the printed words were non-words that were consistent or not, with the inconsistency due to the presence of a SLE (e.g., pocra vs. pocrat). During learning, the incidental presence of an SLE was detrimental to monolinguals but had a facilitative effect on oral vocabulary learning for bilinguals, but these effects were no longer significant 1 day later. Notwithstanding this advantage, both groups stumbled similarly on SLEs when asked to spell the non-words the next day because omissions and substitutions of the SLE accounted for 95 and 93% of spelling errors, respectively. The latter findings, along with those of the present study, suggest that even for multilingual children, constructing orthographic representations of SLEs is difficult. Future research should continue to explore how the relations among oral language, morphological knowledge and spelling might differ between mono- and multilingual children.

## Conclusion

French abounds with silent-letter endings, presenting a substantial challenge for children learning to spell. However, when the SLE contains morphological information, as morphogrammes do, then children’s morphological knowledge may provide recourse, as they can consider derived members of a word family when recalling the root word’s ending. The present study replicated prior findings that morphological knowledge provides unique benefits to children’s morphogramme spelling, as well as provided a thorough examination of the construct of morphological knowledge. Furthermore, the present study explored an avenue for new research into children’s overgeneralization of SLEs. Overall, the present study expands upon our understanding of morphological knowledge and SLE spelling in young elementary children and highlights the importance of including multiple measures when the construct of interest, such as morphological knowledge, is sensitive to developmental change.

## Data Availability Statement

The datasets generated for this study are available on request to the corresponding author.

## Ethics Statement

The studies involving human participants were reviewed and approved by Carleton University’s Research Ethics Boards. Written informed consent to participate in this study was provided by the participants’ legal guardian/next of kin.

## Author Contributions

RM planned and conducted the analyses, and she wrote the first draft of the manuscript. MS and VR initiated the study. VR designed the morpho-phono decomposition task, commented on the penultimate version and approved the final version. MS designed the remaining tasks, supervised the data collection and edited all subsequent versions of the manuscript.

### Conflict of Interest

The authors declare that the research was conducted in the absence of any commercial or financial relationships that could be construed as a potential conflict of interest.

## Appendix

### Decomposition Task (and Correct Answer)

*Training items*: amical (ami); serpentin (serpent); chocolatier (chocolat).

*Test items*: villageois (village; ville); cuisinier (cuisine); écriture (écrit); horloger (horloge); érablière (érable); oreiller (oreille); animalerie (animale); pommier (pomme); naturel (nature); chevalier (cheval); épicier (épice); droitier (droit); marchandise (marchand); juteux (jus); lentement (lent); bavarder (bavard); lignée (ligne); bouffonnerie (bouffon); porcherie (porc); fabricant (fabrique).

### Morpho-Phono Decomposition Task (Phonological Answer; Morphological Answer)

*Training items*: dentier (et; dent); requin (no smaller word = nsw).

*Suffixed test items*: droitier (roi, et; droit), faiblir (lire; faible), fermier (fer, et; ferme), griffure (gris; griffe), impropre (un; propre), impure (un, pue; pure), planchette (plan; planche), plombier (et; plomb), rebond (on; bond), rebord (or; bord).

*Unaffixed test items*: achat (a, chat; nsw), bouteille (bout; nsw), café (fée; nsw), canon (cane, non; nsw), caverne (cave, ver, verre; nsw), cochon (on, coche; nsw), coquille (coq, quillet; nsw), jurer (jus; nsw), pardon (par, don; nsw), profil (pro, prof, fil; nsw).

### Analogy (and Correct Answer)

*Training items:* chien: chienne; chat: (chatte); content: contente; joyeux: (joyeuse); deux: deuxième; trois: (troisième).

*Test items:* gris: grise; blond: (blonde); parfait: parfaitement; heureux: (heureusement); retard retarder; souhait: (souhaiter); méchant: méchante; doux: (douce); jaloux: jalouse; délicat: (délicate); défait: défaite; compris: (comprise); canard: canardeau; souris: (souriceau); grand: grandeur; lent: (lenteur); drap: draperie; tapis: (tapisserie); laid: laideur; épais: (épaisseur); surpris: surprise; parfait: (parfaite); blanc: blanche; froid: (froide); content: contente; bavard: (bavarde); étroit: étroite; sourd: (sourde); haut: hauteur; gros: (grosseur); dent: dentier; dos: (dossier); chaud: chaudement; gratuit: (gratuitement); regard: regarder; aliment: (alimenter); renard: renardeau; éléphant: (éléphanteau); ment: menteur; vend: (vendeur).

### Silent-Letter Ending Orthographic Recognition Task (Correct Answers in Italic)

**Table:**

1.	*chocolat*	chocolas	chocola	11.	repo	repot	*repos*	21.	habid	*habit*	habi
2.	matela	*matelas*	matelat	12.	argumend	*argument*	argumen	22.	*ragoût*	ragoûx	ragoû
3.	tapi	*tapis*	tapit	13.	diaman	diamand	*diamant*	23.	charios	chario	*chariot*
4.	*retard*	retar	retart	14.	*canot*	cano	canop	24.	transpord	*transport*	transpor
5.	ran	*rang*	rant	15.	spor	spord	*sport*	25.	torren	*torrent*	torrend
6.	combas	*combat*	comba	16.	plon	*plomb*	plont	26.	*milliard*	milliar	milliart
7.	*biscuit*	biscuis	biscui	17.	galot	galo	*galop*	27.	fusit	fusi	*fusil*
8.	aimand	aiman	*aimant*	18.	*serpent*	serpen	serpend	28.	*avocat*	avocas	avoca
9.	sabo	sabop	*sabot*	19.	*confort*	conford	confor	29.	dra	*drap*	drat
10.	*lézard*	lézar	lézart	20.	documen	documend	*document*	30.	*outil*	outit	outi

### General Orthographic Recognition Task (Correct Answers in Italic)

**Table:**

1.	*graisse*	graice	11.	*jambe*	jembe	21.	baucal	*bocal*
2.	orteille	*orteil*	12.	fain	*faim*	22.	painture	*peinture*
3.	bombon	*bonbon*	13.	*sauce*	sausse	23.	jirafe	*girafe*
4.	*quille*	qille	14.	cinture	*ceinture*	24.	*brosse*	broce
5.	*garder*	guarder	15.	*menton*	manton	25.	*baleine*	balaine
6.	tanbour	*tambour*	16.	sommeille	*sommeil*	26.	flaucon	*flocon*
7.	*frein*	frain	17.	guardienne	*gardienne*	27.	*japper*	japer
8.	guarderie	*garderie*	18.	*magasin*	magazin	28.	trein	*train*
9.	naige	*neige*	19.	*trésor*	trézor	29.	bule	*bulle*
10.	*frapper*	fraper	20.	*rappel*	rapel	30.	*reine*	raine

### Decoding Task (Correct Answers in Italic)

**Table:**

1.	*fraze*	traze	11.	umin	*ubin*	21.	*geamès*	geomès
2.	*phrès*	drès	12.	*amphant*	amdant	22.	daremps	*paremps*
3.	harfé	*harmé*	13.	loce	*lèce*	23.	*hinoscent*	binoscent
4.	*kuillaire*	tuillaire	14.	*geois*	geoif	24.	*genvié*	genrié
5.	réamp	*jéamp*	15.	aksidont	*aksidant*	25.	cefrait	*cekrait*
6.	liot	*liom*	16.	mahibe	*mahisse*	26.	açanfeur	*açanceur*
7.	*rekin*	tekin	17.	fainke	*sainke*	27.	*geardaim*	gearlaim
8.	*éguiye*	éguipe	18.	*rèzon*	rèton	28.	*rèzim*	rèmin
9.	sate	*saje*	19.	mévrié	*phévrié*	29.	muque	*duque*
10.	*movet*	mopet	20.	*seinje*	feinje	30.	katé	*karé*
